# Endothelin-converting enzyme-1 expression in acute and chronic liver injury in fibrogenesis

**DOI:** 10.1080/19768354.2019.1595141

**Published:** 2019-06-09

**Authors:** Tae-Jun Cho, Hyo-Jung Kim, Jaejin Cho

**Affiliations:** a Labratory of Developmental Biology and Stem Cell Differentiation/Transplantation, Department of Dental Regenerative Biotechnology, School of Dentistry, Seoul National University South Korea, Seoul, South Korea; b Dental Research Institute, Seoul National University, Seoul, South Korea

**Keywords:** Endothelin-converting enzyme-1, endothelin-1, hepatic fibrogenesis, endothelin receptor

## Abstract

Endothelin-1 (ET-1) induces contraction, proliferation, and collagen synthesis of activated hepatic stellate cells and is a potent mediator of portal hypertension. Endothelin-converting enzyme-1 (ECE-1) generates ET-1 from the inactive precursor big-endothelin-1. The cellular distribution and activity of ECE-1 in the liver is unknown. Hepatic fibrogenesis was induced in rats by CCl_4_ administration and secondary biliary cirrhosis after 6 weeks of complete bile duct occlusion (BDO). The tissue ET-1 and ET receptor protein levels were quantified, the ECE-1 isoform mRNAs were measured by RNase protection assay and ECE-1 activity was analyzed. ECE-1a and -b mRNA were upregulated in biliary cirrhosis and in CCl_4_-injured livers, whereas ECE-1c mRNA remained unchanged. ECE-1 activity was increased after BDO and peaked at 12 h after acute CCl_4_-intoxication. Tissue levels of ET-1, ET_A_- and ET_B_ receptors were elevated 7-, 5-, and 4.6-fold in cirrhotic rats, respectively. ECE-1 activity increased following BDO and acute CCl_4_-intoxication. In conclusion, ECE-1a and -b RNAs are upregulated in fibrogenesis, indicating that these isoforms play a central role in ET-1 generation during fibrogenesis and portal hypertension.

## Introduction

Endothelins (ETs) comprise a family of three homologous 21-amino acid oligopeptide vasoactive mediators (ET-1, ET-2, and ET-3) that trigger various biological effects through G protein-coupled specific receptors, namely the ET_A_ receptor and ET_B_ receptor (ET_A_R and ET_B_R, respectively). ET-1 usually induces a long-lasting vasoconstriction *via* ET_A_R and vasodilation *via* ET_B_R (Barman 2007; Martell et al. 2010; Ling et al. 2012).

The biological precursor of ET-1, prepro-ET-1, is converted to biologically active ET-1 through two steps. Initial cleavage of two basic amino acids by a furin-like enzyme results in big-ET-1, which is further processed by cleavage of a Trp-Val bond by endothelin converting enzyme-1 (ECE-1) producing biologically active ET-1. ECE-1 is a membrane-bound, phosphoramidon-sensitive metalloproteinase (Rodriguez-Pascual et al. 2014).

**Figure 1.**
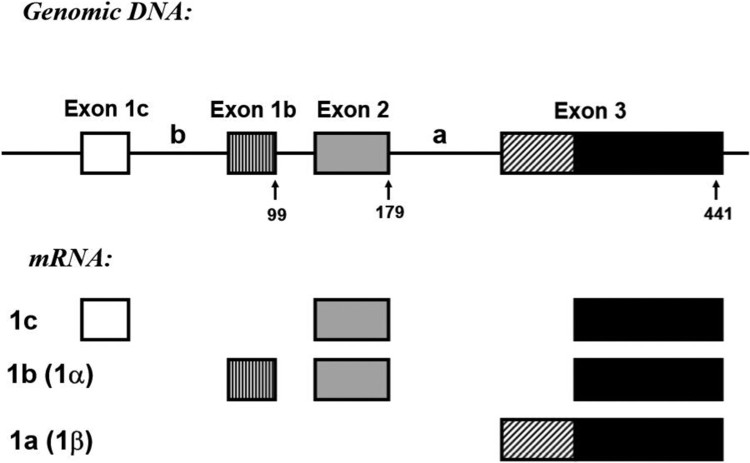
Genomic structure and mRNA isoforms generated from the rat ECE-1 gene. Note: The genomic structure of the 5′-terminal part of the rat ECE-1 gene with exon-intron boundaries and localizations of the alternative promoters is shown in the upper part. Exons 4–19, which are common to all isoforms, are not shown. ECE-1 isoforms 1a, 1b, and 1c transcribed by different promoters are shown in the lower part.

Several ECE-1 isoforms have been identified (Figure 1). ECE-1b and -c are characterized by isoform-specific exon 1, which are spliced to exon 2 that is common to both of these isoforms. ECE-1a is transcribed from an alternative promoter located about 11 kb downstream of exon 2. The 5′-terminus of exon 3 is the first ECE-1a specific exon, whereas the 3′-terminal part of this exon is common to all ECE-1 isoforms (Valdenaire et al. 1995; Orzechowski et al. 1997; Schweizer et al. 1997). Furthermore, an additional isoform that is generated from exon 1d located within the small (approx.. 200 bp) genomic region between exon 1b and exan 2, ECE-1d, was recently identified (Muller et al. 2003). ECE-1 is upregulated in congestive heart failure (Kohan et al. 2011), gastric injury (Slomiany and Slomiany 2005) and buccal mucosal ulcer healing after chronic alcohol ingestion in rats (Slomiany et al. 2000), and in lung cancer (Moody et al. 2017).

In the liver, the endothelin system is a modulator of portal hypertension and fibrogenesis. Mediated through the ET_A_R, ET-1 stimulates proliferation and contraction of hepatic stellate cells (HSC). This profibrogenic effect was significantly ameliorated in a model of rat biliary fibrosis following bile duct occlusion (BDO) by treatment with an orally active, highly specific ET_A_R antagonist, as evidenced by reduced hepatic collagen accumulation (Cho et al. 2000). As a key enzyme to the ET-1/ET receptor-system, ECE-1 and its isoforms may constitute additional targets for pharmacological intervention. A recent study suggested that ECE-1 transcripts were downregulated, whereas ECE-1 protein expression was increased in hepatic stellate cells isolated from fibrotic rat livers. Furthermore, the activity of ECE-1 did not change after liver injury induced by carbon tetrachloride and bile duct ligation in rats (Hocher et al. 2011). However, neither cellular sources nor expression levels of ECE-1 or its isoforms were studied *in vivo*. Therefore, we investigated the temporospatial expression patterns of ECE-1 at the RNA level by *in situ* hybridization and isoform-specific RNase protection assays and related these to ECE-1 activity in acute and chronic liver fibrogenesis.

## Material and methods

### Animals and experimental design

All animal experiments were conducted in accordance with German state laws approving and governing the use of experimental animals. Ten week old female Wistar rats (Schoenwalde, Germany., approx. 250 g) were maintained under 12-hour light–dark cycles and at 23 ± 2°C with a humidity of 60 ± 10%. During the experiment, the animals received pellet chow diet freely (Ssniff, Germany). The rats were used for induction of secondary biliary fibrosis by complete bile duct occlusion (BDO) and scission following retrograde injection of Ethibloc (sodium amidotrizate) as previously described (Cho et al. 2000; Karsdal et al. 2016; Santos-Laso et al. 2017). Animals were sacrificed after 6 weeks when tissue hepatic collagen is increased 8–12 fold served as controls. Acute fibrogenesis was induced in 7 week old male rats (weight approx. 125 g) by a single dose of carbon tetrachloride (CCl_4_) (Sigma, St. Louis, MO, USA) injected intraperitoneally at 1.25 ml/kg body weight and sacrificed in groups of 6, 12, 24, 48, and 72 h animals.

### Determination of hepatic ET-1, and of ET_A_ and ET_B_ receptor levels

Analysis of tissue ET-1 concentration was conducted as previously described (Cho et al. 2000; Kocyigit et al. 2018). Briefly, Snap-frozen liver tissue (approx. 200 mg) was powdered in liquid nitrogen and homogenized at 4°C. Homogenates were then centrifuged at 4°C for 60 min at 100,000 × g, after which supernatants were analyzed for ET-1 content using the commercial enzyme immunoassay (Biomedica, Vienna, Austria). Cross-reactivities were as follows: ET-1 (1–21): 100%; ET-2 (1–21): 100%; ET-3 (1–21): < 5%; big-ET-1 (1–38): < 1%; big-ET-2 (22–38): < 1%. ET receptors were determined by radioligand assays (Cho et al. 2000) in the presence or absence of the subtype-specific endothelin receptor antagonists BQ123 (3 µmol/l, ET_A_R specific) (Sigma, St. Louis, USA) and/or BQ3020 (3 µmol/l, ET_B_R specific) (Sigma, St. Louis, USA). Next, 1 ml of cold binding buffer was added and centrifugated at 30,000 × g and 4°C for 15 min and receptor-bound [^125^I]-ET-1 was counted in a Packard Gamma Counter with 78% counting efficiency for [^125^I].

## Cloning of cDNA probes and multiprobe RNase protection assay

The cDNA for rat endothelin converting enzyme-1 (ECE-1) covering coding positions 1–441 bp was prepared from rat liver tissue by RT–PCR amplification according to the published sequence (Shimada et al. 1994) (GenBank accession number: D 29683, sense primer: TGCGGTCGGAGCGTAGAGCT, antisense primer: ACCACAGGCGTAGCTGAAGAA), verified by sequence analysis and cloned into pZErO-1 (Invitrogen, San Diego, CA, USA). The probe was designed to detect the three different ECE-1 isoforms (ECE-1a, b, and c). The protected sequences were as follows: ECE-1a, 260 bp; ECE-1b, 340 bp; ECE-1c, 439 bp (Figure 1). Internal standardization was conducted using a 102 bp GAPDH probe (from position 335 to 437, M17701). Preparation of total RNA and RNase protection assays were performed as described previously (Cho et al. 2000). Briefly, after extraction of RNA using acid guanidinium thiocyanate-phenol–chloroform, the integrity of all samples was documented by visualization of 18S and 28S ribosomal bands after electrophoresis. Radiolabeled cRNA was produced by *in vitro* transcription with T7 polymerase (Ambion, Austin, TX, USA) using [α-^32^P] UTP (800 Ci/mmol, 10 mCi/mL; NEN, Boston, MA, USA), followed by incubation with 10^5 ^cpm of ^32^P-labeled cRNA, denaturation at 95°C and overnight hybridization at 42°C. Following hybridization, RNase A and T1 (Ambion, Austin, TX, USA) digestion of unbound labels and unprotected mRNA was conducted. The protected RNA-RNA hybrids were denatured and separated by electrophoresis through a 5% polyacrylamide/8M urea sequencing gel, after which the gel was exposed to an X-ray film for 48 h. Autoradiograms were analyzed with the public domain NIH Image program. Signals for ECE-1 isoform mRNAs were normalized to the signal for GAPDH mRNA and expressed as relative abundance (arbitrary units). The differences in relative abundance of mRNAs were analyzed by the Kruskal–Wallis test.

## Endothelin-converting enzyme activity

Endothelin-converting enzyme activity was measured by the production rate of endothelin-1 from big-endothelin with minor modifications of a previously reported method (Mitani et al. 2000). Briefly, membrane-bound proteins were enriched by homogenization of 100 mg liver tissue in 1 ml 20 mM Tris-HCl, pH 7.4, containing 5 mM MgCl_2_, 20 µM pepstatin A, 20 µM leupeptin, and 50 µM *p*-amidinophenyl methanesulphonyl fluoride (homogenization buffer). The homogenates were then centrifuged at 1,000 *g* and 4°C for 10 min to remove cell debris, followed by high-speed centrifugation of 200 µl of the supernatant at 100,000 *g* and 4°C for 45 min. The resultant pellets were solubilized in homogenization buffer containing 0.5% (w/v) Triton X-100 and centrifuged again (100,000 *g*, 4°C for 60 min), after which the supernatant was transferred to fresh tubes. Next, 25 μL of the supernatant was diluted with 100 µL assay buffer (20 mM Tris-HCl, pH 6.8, containing 100 nM big endothelin-1, 0.1% (w/v) bovine serum albumin, 20 µM pepstatin A, 20 µM leupeptin) and incubated for 2 h at 37°C. The reaction was stopped with 125 µL 5 mM EDTA and the concentration of generated ET-1 determined with the endothelin ELISA kit as described above. This was normalized to protein concentration as measured by the Micro BCA assay kit (Pearce, Rockford, IL, USA).

## Statistics

Statistical analysis was performed using analysis of variance, Duncan's multiple range test for differences between groups, and Pearson's correlation coefficient.

## Results

### Tissue endothelin-1 and endothelin receptor protein tissue levels

**Figure 2.**
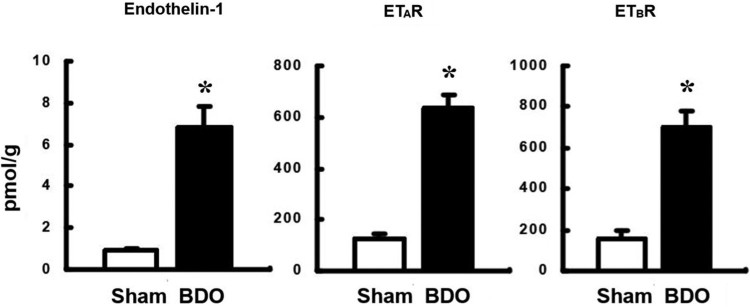
Upregulation of tissue endothelin-1 and endothelin A and B receptor levels in rats with secondary biliary cirrhosis. Note: BDO, bile duct occlusion; Sham, sham operation as a negative control. ETAR, endothelin A receptor; ETBR, endothelin B receptor. *, *P *<  0.05 vs. control.

Hepatic levels of ET-1, ET_A_R and ET_B_R were increased 7.2-, 7.35- and 4.9-fold, respectively, in BDO rats compared to the normal control group (*p* < 0.001), indicating a strong activation of the endothelin system in chronic liver fibrogenesis (Figure 2).

### Patterns of ECE-1 mRNA expression in normal liver and in hepatic fibrogenesis

In situ hybridization with [^35^S]-labeled RNA probes was used to define the cellular source of ECE-1 expression. In normal liver, ECE-1 transcript levels were homogeneously distributed over hepatocytes. Some perisinusoidal nonparenchymal cells also expressed ECE-1 RNA at levels similar to hepatocytes. Slightly increased signals were found over endothelial cells of the portal and terminal hepatic veins and arteries. Occasionally, bile duct epithelia showed low-level ECE-1 RNA expression.

Compared to normal liver cells, all hepatic cells displayed increased ECE-1 transcript levels in biliary cirrhosis. Hepatocytes as well as perisinusoidal cells were the major locations of ECE-1 mRNA expression, whereas ECE-1 specific autoradiographic signals remained lower in bile duct cells and portal fibroblasts.

At 24 h after injection of CCl_4_, ECE-1 transcript levels increased over perisinusoidal as well as central/portal venular and arterial endothelial cells. Some of these cells were desmin positive, indicating they were activated stellate cells or myofibroblasts. As in the BDO model of chronic fibrogenesis, ECE-1 RNA predominated in hepatocytes. Desmin-positive cells adjacent to pericentral areas of necrosis showed higher levels of ECE-1 mRNA than hepatocytes, whereas bile duct epithelia displayed lower ECE-1 RNA levels compared to hepatocytes.

### Expression of ECE-1 isoform mRNAs in acute and chronic hepatic fibrogenesis

**Figure 3.**
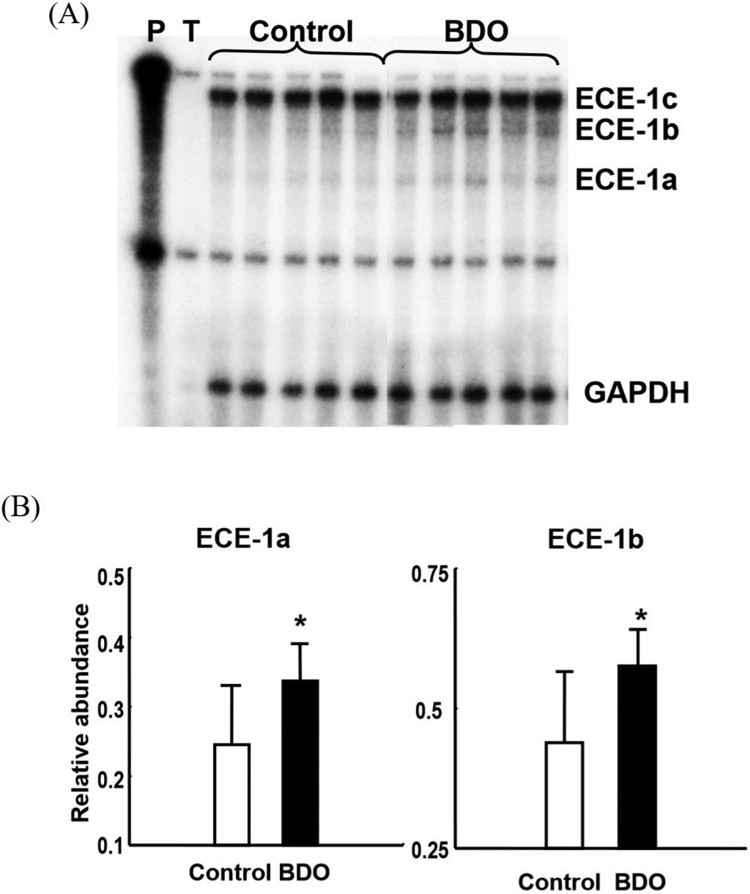
Upregulation of endothelin converting enzyme-1 a and b, but not c isoform mRNA in rats with secondary biliary cirrhosis. Note: Multiprobe-RNase protection assay. (A) Autography and (B) relative abundance of endothelin converting enzyme-1 a and -1 b isoform mRNAs. P, cRNA probes, T, negative control containing transfer RNA; Control, normal liver; BDO, BDO alone as positive control; *, *P *<  0.05 vs. control.

**Figure 4.**
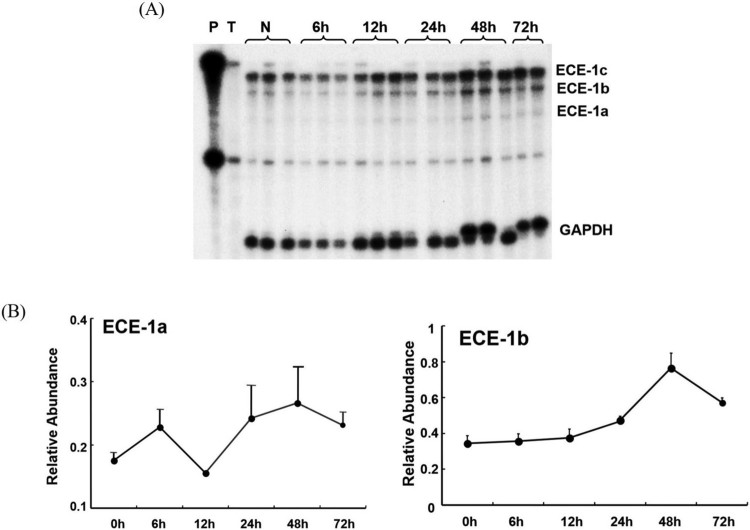
Dynamics of endothelin converting enzyme-1 isoform mRNAs expression in rats with acute carbon tetrachloride intoxication. Note: Multiprobe-RNase protection assay. (A) Autography and (B) relative abundance of endothelin converting enzyme-1 a and -1 b isoform mRNAs. P, cRNA probes, T, negative control containing transfer RNA; N, normal liver.

To differentiate the expression patterns of the three ECE-1 isoform mRNAs (ECE-1a, -1b and -1c), an ECE-1 cDNA probe that allowed detection of the three isoforms during the RNase protection assay was generated. After 6 weeks of BDO as well as after a single dose of CCl_4_, expression of the predominant ECE-1c isoform remained unchanged. However, ECE-1a and -1b isoform mRNAs were increased slightly by 1.3–1.4 fold in biliary fibrosis and 1.5–2.2 fold at 48 h after CCl_4_-induced acute liver injury (*p* < 0.05), respectively (Figure 3). After CCl_4_ intoxication, both ECE-1a and -1b isoforms increased in a time-dependent manner, with the maximum occurring after 48 h (Figure 4).

### ECE-1 activity in biliary fibrosis and after acute CCl_4_ intoxication

**Figure 5.**
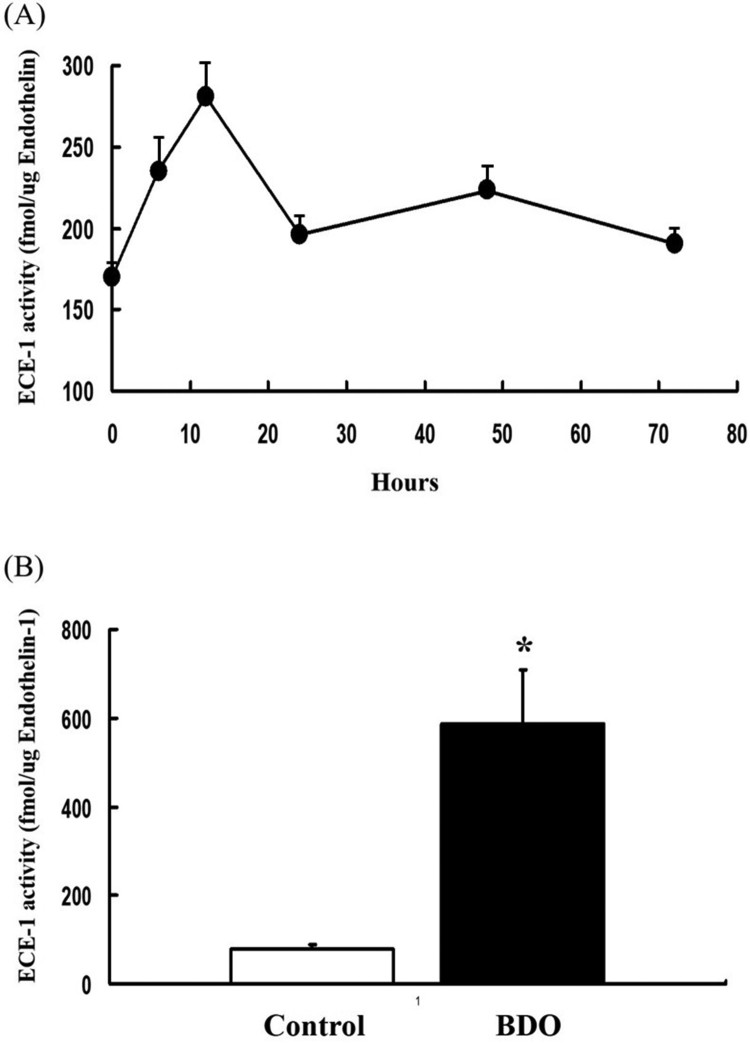
Endothelin-converting enzyme-1 activity. Note: Conversion of big ET-1 to ET-1 by ECE-1 in solubilized membrane fractions of rat liver tissues. (A) ECE-1 activity in acute liver injury induced by carbon tetrachloride and (B) in advanced liver fibrosis due to bile duct occlusion for 6 weeks by rat secondary biliary fibrogenesis.

We measured the activity of ECE-1 to clarify whether the increases in ECE-1a and -1b isoform mRNAs were coupled with enhanced ECE-1 enzymatic activity, as reflected by increased *in vivo* generation of ET-1. In biliary fibrosis, ECE-1 activity increased 7-fold compared to the normal controls (*p* < 0.05) (Figure 5). After acute CCl_4_ intoxication, ECE-1 activity (Figure 5(B)).

## Discussion

The endothelin system plays an important role in vascular homeostasis and various pathophysiological processes, including liver diseases. Thus, it is activated in cardiovascular diseases (Kalk et al. 2011) and atherosclerosis (Huang 2009), pulmonary disease (Moody et al. 2017) and hepatic fibrosis (Cho et al. 2000). ECE-1, of which there are at least four isoforms, is the key enzyme involved in ET-activation and may play an important role in hepatic fibrogenesis and other diseases. Because little is known about ECE-1 expression in the liver, we investigated the expression patterns of ECE-1 and its isoforms during acute and chronic hepatic fibrogenesis.

Because tissue levels of ET-1, ET_A_R and ET_B_R are upregulated during liver fibrogenesis while the ET-1 processor big-ET-1 remains unchanged (Figure 2), ECE-1 should serve as the gatekeeper for enhanced ET production.

We found that ECE-activity increased by 1.3–1.4 fold in BDO and after acute (CCl4-induced) fibrogenesis. This was reflected by moderate, up to 1.3 and 1.4 fold increased expression of hepatic ECE-1a and -1b mRNA, respectively, whereas ECE-1c mRNA remained unchanged. Because parenchymal ECE-1 mRNA was constitutive constitutive and that by endothelial as well as some desmin-positive (myofibroblastic) cells upregulated, it may be speculated that ECE-1a and -1b isoforms are mainly produced by nonparenchymal cells and upregulated during fibrogenesis.

Our results are in accordance with those of previously conducted studies. For example, tissue ECE activity is correlated with coronary artery disease (Nguyen et al. 2010), while ECE-1 activity was show to be upregulated during buccal mucosal ulcer (Slomiany et al. 2000) and gastric ulcer (Slomiany and Slomiany 2005) in rats and accompanied by the induction of TNF-α and apoptosis. Furthermore, inhibition of ECE-1 attenuated atherosclerosis (Grantham et al. 2000) and hypertension (Takeda et al. 2000). Summarizing those results indicates that the ET-system, especially ECE, might play a crucial role in the pathophysiology of tissue regeneration. Similarly, our data confirmed that ECE is of great importance to liver fibrogenesis. However, the results of the present study contradicted those of Shao et al., who showed that the ECE-1 mRNA level in hepatic stellate cells was reduced and ECE-1 protein content was increased in liver fibrosis (Nagata et al. 2005; Hocher et al. 2011). These differences likely occurred because of differences in the experimental model. Specifically, Shao et al. used freshly isolated hepatic stellate cells, whereas we used whole liver tissues. Because ECE-1 mRNA expression was widely distributed in not only HSC, but also hepatocytes, perisinusoidal cells, and venular/arterial endothelial cells, the action of ECE-1 is not limited to HSC. Furthermore, the different expression patterns depend on ECE-1 isoforms.

Overall, the results of this study indicate that modulation of ECE-1 isoform expression might play a key role in ET-system activation in liver fibrogenesis.
